# Morphologic characterization of osteosarcoma growth on the chick chorioallantoic membrane

**DOI:** 10.1186/1756-0500-3-58

**Published:** 2010-03-04

**Authors:** Maurice Balke, Anna Neumann, Christian Kersting, Konstantin Agelopoulos, Carsten Gebert, Georg Gosheger, Horst Buerger, Martin Hagedorn

**Affiliations:** 1 Department of Orthopaedic Surgery, University of Muenster, Muenster, Germany; 2 Gerhard-Domagk-Institute of Pathology, University of Muenster, Muenster, Germany; 3 Institute of Pathology, Paderborn, Germany; 4 Department of Orthopaedic Surgery, Orthopädische Klinik Volmarstein, Wetter, Germany; 5 INSERM U920 - Laboratoire des mécanismes moléculaires de l'angiogenèse, University Bordeaux 1, Talence cedex, France

## Abstract

**Background:**

The chick chorio-allantoic membrane (CAM) assay is a commonly used method for studying angiogenic or anti-angiogenic activities *in vivo*. The ease of access allows direct monitoring of tumour growth by biomicroscopy and the possibility to screen many samples in an inexpensive way. The CAM model provides a powerful tool to study effects of molecules, which interfere with physiological angiogenesis, or experimental tumours derived from cancer cell lines. We therefore screened eight osteosarcoma cell lines for their ability to form vascularized tumours on the CAM.

**Findings:**

We implanted 3-5 million cells of human osteosarcoma lines (HOS, MG63, MNNG-HOS, OST, SAOS, SJSA1, U2OS, ZK58) on the CAM at day 10 of embryonic development. Tumour growth was monitored by *in vivo *biomicroscopy at different time points and tumours were fixed in paraformaldehyde seven days after cell grafting. The tissue was observed, photographed and selected cases were further analyzed using standard histology.

From the eight cell lines the MNNG-HOS, U2OS and SAOS were able to form solid tumours when grafted on the CAM. The MNNG-HOS tumours showed the most reliable and consistent growth and were able to penetrate the chorionic epithelium, grow in the CAM stroma and induce a strong angiogenic response.

**Conclusions:**

Our results show that the CAM assay is a useful tool for studying osteosarcoma growth. The model provides an excellent alternative to current rodent models and could serve as a preclinical screening assay for anticancer molecules. It might increase the speed and efficacy of the development of new drugs for the treatment of osteosarcoma.

## Introduction

Osteosarcoma is the most common primary malignant bone tumour that usually affects children and adolescents. After the introduction of cytotoxic polychemotherapy to the treatment of osteosarcoma tremendous advances were achieved, increasing the survival rate from under 20% to over 60%[1]. But over the past 20 years no substantial improvements were made. There is still a high failure rate especially when metastases are present[2]. It is therefore of critical importance to identify new targets and new molecules with efficient antitumour activity. New targets might be identified by large-scale microarray analysis or array-based genomic hybridization[3] to identify different gene expressions in patient samples or suitable *in vivo *animal models. It is expected that anti-angiogenic agents will have clinical benefits for patients with osteosarcoma, probably by reducing metastasis[4]. Thus especially models that allow precise monitoring of tumour angiogenesis might be promising in finding new targets to treat osteosarcoma[5].

An *in vivo *assay to serve these purposes is the chick chorio-allantoic membrane (CAM) assay. The CAM is formed on embryonic day 4 - 5 by fusion of the chorion with the allantoic vesicle[6]. The resulting membrane consists of two mesodermal layers: the somatic mesoderm of the chorion and the splanchnic mesoderm of the allantois. In this structure, an extremely dense vascular network develops with bigger vessels located within the mesoderm and the capillaries situated directly under or within a very thin ectodermal layer. The physiological role of the CAM is to serve as a respiratory organ until hatching, to store waste products and to absorb calcium from the shell for bone development[7]. On the molecular level critical genes which participate in the vascularisation of complex human tissues such as placenta and the lung are significantly regulated during the CAM vascular development[8]. The CAM assay is characterized by several major advantages such as the ease of access, the extensive vascularisation and the relatively simple experimental approach. This opens up the possibility to screen many samples in an inexpensive way[9].

Since its use to demonstrate normal embryonic blood vessel development more than 100 years ago, the CAM served as a host for transplantations of embryonic tissue as well as for bacteria and viruses. During the last 20 years the CAM is extensively used in angiogenesis research but a reliable bone tumour model has not yet been reported.

Here we present the results of the screening of the human osteosarcoma cell lines HOS, MG63, MNNG-HOS, OST, SAOS, SJSA1, U2OS and ZK58 for their use in the CAM assay and provide evidence that the MNNG-HOS cell line reproducibly simulates key features of human osteosarcoma growth.

## Materials and methods

### Cell culture

Eight osteosarcoma cell lines (HOS, MG63, MNNG-HOS, OST, SAOS, SJSA1, U2OS, ZK58) were used in this study. All cell lines were cultured in RPMI 1640 (E15-840, PAA Austria) supplemented with 10% Fetal Bovine Serum (FBS Gold, A15-649, PAA Austria) and 1% penicilline/streptomycine (P11-010, PAA Austria) at 37°C in a humidified 5% CO_2 _incubator. Prior implantation, cell suspensions were prepared by detaching cells with trypsin/EDTA (L11-004, PAA Austria). Cells were centrifuged at 1200 rpm for 5 min, washed twice in culture medium and resuspended in medium at a final concentration of 3 to 5 million cells per 20 μl[10].

### CAM assay

Fertilized white leghorn chicken eggs (Lohmann Tierzucht GmbH, Cuxhaven, Germany) were incubated at a humidity of 70% and 37°C. On day 3 of incubation, a round window was cut into the shell after removal of 2 - 3 ml of albumen allowing detachment of the embryo from the eggshell. Normal development was verified and embryos with malformations or dead embryos were excluded. The window was sealed with tape and the eggs were returned to the incubator. On day 10 of development, small plastic rings made out of Thermanox™ discs were placed on the CAM and 25 μl of medium containing different cell lines were deposited into the rings after gentle laceration of the CAM surface. The number of CAMs implanted for each cell line is indicated in Table 1. CAMs were examined daily until day 17 and photographed *in ovo *with a digital camera (Olympus E330) attached to a stereomicroscope. CAMs were checked for tumour growth. Positive tumour development ("+" tumours) was reported when tumour angiogenesis was visible and when tumours were bigger than 2 mm. All other tumours were classified as ("-"). The estimated tumour volume was calculated (according to Hagedorn et al 2005[10]) by the following formula: V = 4/3* π *r^3 ^(r = 1/2 * square root of diameter 1 * diameter 2). Frequently several independent tumour nodules developed. However for quantification only the biggest nodule was taken. Only CAMs still alive at day 17 were included in this analysis.

**Table 1:** Overview of tested osteosarcoma cell lines

Cell line	Number of grafted CAMs	Embryos died	Number of - tumours	Number of + tumours	Ratio +/-	% dead	*P*-value*
MNNG-HOS	23	6	6	11	11/17	26.1	*NS*
U2OS	21	11	5	5	5/10	52.4	0.0294
SAOS	20	14	3	3	3/6	70	0.0008
OST	8	2	5	1	1/6	25	*NS*
MG63	14	7	4	1	1/7	50	*NS*
HOS	20	5	13	2	2/15	25	*NS*
ZK58	35	11	21	3	3/24	31.4	*NS*
SJ-SA-1	11	2	9	0	0/9	18.2	*NS*
Controls	26	5				19.2	

n = number, - = no tumour/tumour ≤ 2 mm, + = vascularized tumour > 2 mm

*Fisher's exact test (cell line vs. controls); *NS *= not significant

### Statistical analysis

To determine if osteosarcoma growth on the CAM was associated with increased embryonic death, a 2 × 2 contingency table test (Fisher's exact test) was used for each cell line compared to control CAMs.

### Histology

On embryonic day 17, a few ml of 4% paraformaldehyde were put onto the CAM after photo documentation. After 20 min the window was carefully enlarged using scissors without destroying the CAM. Areas containing the tumour were cut out and transferred into culture discs. All tumours were evaluated and relevant samples were embedded in paraffin and processed for sectioning. Embedded tumours were cut into 10 μm sections, stained with hematoxylin-eosin and further analysed by standard light microscopy (Leica DM2500 with Leica EC3 camera).

## Results

**Figure 1:**
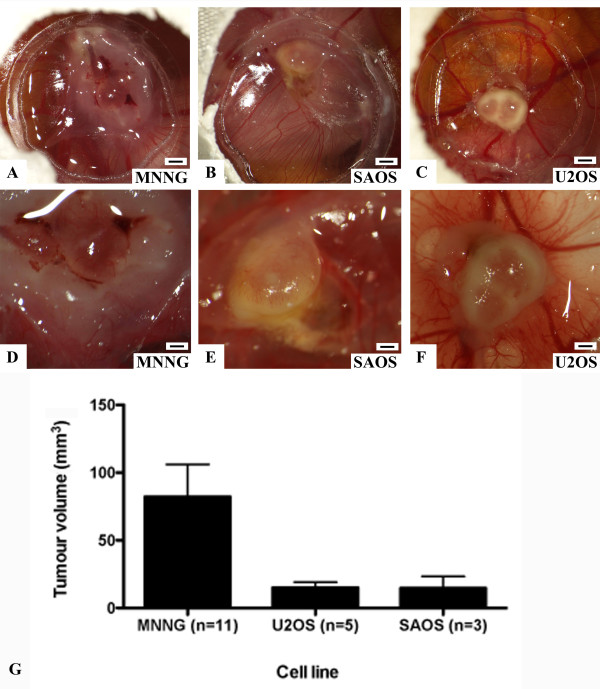
**Tumours of three different cell lines grown on the CAM**. *In vivo *microscopy of tumours grown of indicated cell lines after 7 days of tumour growth. **A **- **C**: magnification 10× (scale bar 1 mm), **D **- **E**: higher magnification (40×, scale bar 250 μm) of **A **- **C**. **G**: Graph illustrating the mean tumour volumes calculated by the following formula: V = 4/3*π*r^3 ^(r = 1/2 * square root of diameter 1 * diameter 2). SEM = standard error of mean.

From the eight cell lines tested only the MNNG-HOS, U2OS and SAOS line consistently (= more than in 50% of the cases alive at day 17) formed vascularized tumours of more than 2 mm in size (Figure 1). In all other cell lines, the tumours where either smaller or did not develop at all (Table 1). Grafting of the U2OS and SAOS cell lines was significantly associated with increased embryonic death, compared to non-grafted CAMs (P-values: 0.0294 and 0.0008, respectively).

**Figure 2:**
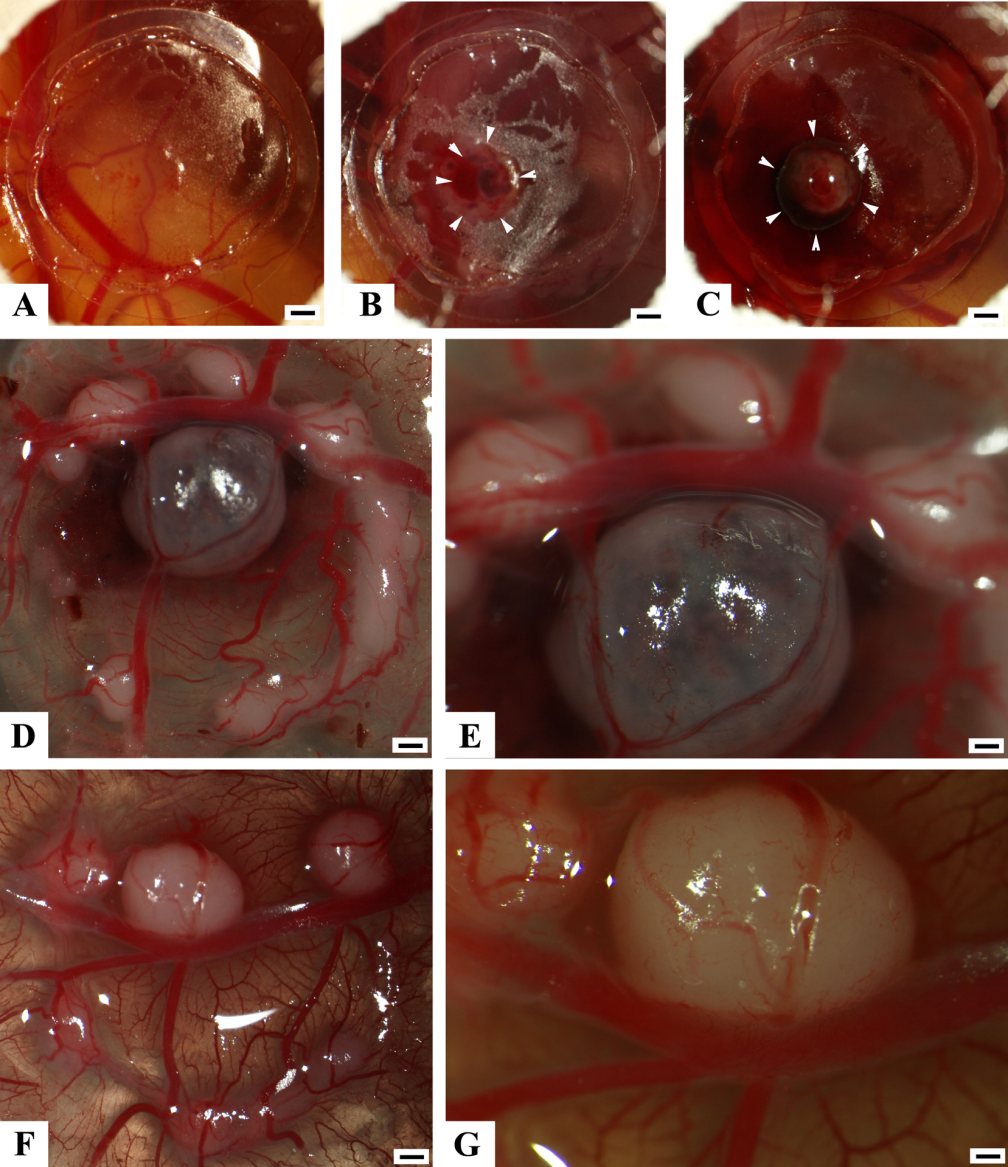
**Typical growth pattern of MNNG-HOS cells grafted to the CAM**. **A **- **C**: *In vivo *microscopy (10×, scale bar 1 mm) documenting the typical growth pattern after implantation of MNNG-HOS cells, arrowheads indicate tumour boundaries. **A **1 day after grafting. After 4 days a vascularized tumour becomes apparent (**B**). Solidification of the tumour steadily progresses until day 7 (**C**). Note the bleeding caused by the tumour in **C**. **D **(12.5×, scale bar 0.8 mm) and **E **(50×, scale bar 20 μm) are higher magnifications of **C **after turning the CAM upside down. Note the three smaller tumour nodules that were not visible from the upside (**C**). **F **(12.5×, scale bar 0.8 mm) and **G **(50×, scale bar 20 μm) is another example of a typical MNNG-HOS tumour. Note the rich vascularization of the tumour (**G**).

MNNG-HOS, U2OS and SAOS tumours revealed a reproducible growth pattern after implantation of the cells. After 4 days, a vascularized tumour became apparent. Growth and vascularization of the tumour steadily progressed until day 7 as evidenced by biomicroscopy (Figure 2A-C). In some cases of MNNG-HOS tumours, growth was associated with bleeding (Figure 2C-E). Furthermore, MNNG-HOS tumours showed an invasive growth pattern and were able to penetrate the CAM and grow underneath it. This was not observed in the U2OS and SAOS tumours that stayed on the surface of the CAM or within the membrane. This became clearly apparent when the membrane was cut out after fixation and turned upside down (Figure 2D-G). The surrounding vessels were attracted towards the tumour tissue. At higher magnification, blood flow could be observed, a direct evidence of the functionality of the tumour capillaries (data not shown). Biomicroscopy of the tumours revealed a very rich vascularisation showing signs of active sprouting angiogenesis (Figure 2G).

**Figure 3:**
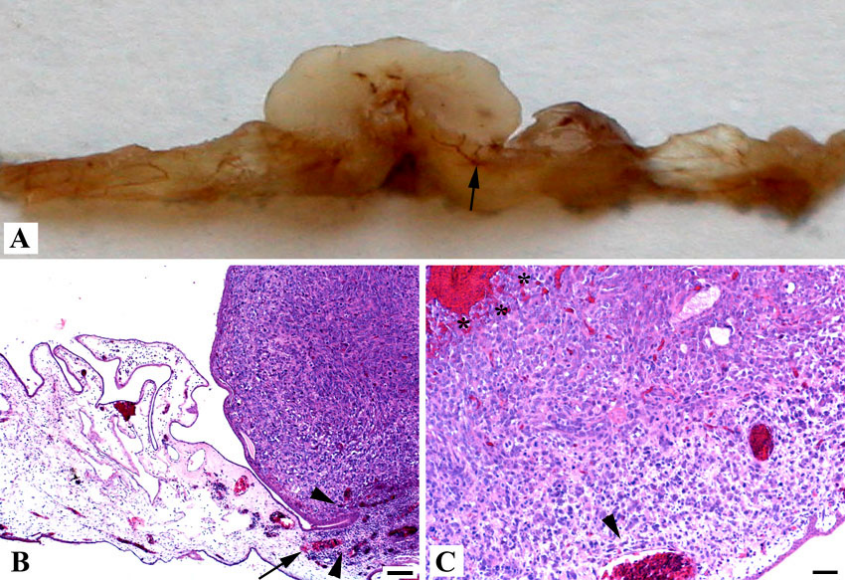
**Standard histology of MNNG-HOS tumour**. Standard histology with hematoxylin-eosin staining confirming the rich vascularisation of the tumours with capillaries originating from the CAM (arrows in **A **and **B**). The tumours also contain stromal cells with disseminated monocytes. Areas of angiogenesis (arrowheads in B and C), necrosis and haemorrhage (asterisks in **C**) are present. **A**: Macroscopic picture, **B**: HE 10× (scale bar 100 μm), **C**: HE 40× (scale bar 50 μm).

Standard histology with hematoxylin-eosin staining confirmed the rich vascularisation of the tumours with capillaries sprouting out of the CAM into the tumour (Figure 3A, B). Nucleated chick erythrocytes were present in the capillaries (Figure 3C). The tumours contained stromal cells with disseminated uncharacterized macrophages. Areas of angiogenesis, necrosis and haemorrhage were present, a typical feature of human osteosarcoma (Figure 3B + C). Osteoid formation did not occur in any cell line. Controls with medium alone did not show any tissue growth, inflammatory reaction or vascular response.

## Discussion

Experimental *in vivo *tumour models are essential for comprehending the dynamic process of human cancer progression, identifying therapeutic targets, and evaluating antitumour drugs. Besides the promising efforts made in the research on osteosarcoma using *in vitro *assays[11] there is a lack of potent and reproducible *in vivo *models to evaluate new therapies in a pre-clinical setting.

The chick chorioallantoic membrane assay is a commonly used method for studying angiogenic or anti-angiogenic activities *in vivo*[12]. Although first tumour transplantations to the CAM were described more than 100 years ago[13] CAM tumour models are still rarely developed and characterized, compared to murine models[14]. Taking into account the easy access, relatively simple experimental approach and the natural immonodeficient environment of the developing embryo (for review see[15]) its rare use for tumour grafting is surprising. Recently, a reliable experimental glioblastoma CAM model using the human U87 cell line has been established[10]. The experimental glioma model simulates key features of human glioma growth in a few days. Treatment with the tyrosine kinase inhibitors Gleevec (imatinib) or PTK787/ZK 222584, siRNA-mediated knock down of Vascular Endothelial Growth Factor (VEGF) and/or IL6[16,17] potently inhibited tumour angiogenesis and growth in this model.

Another group inoculated single cell suspensions of LNCaP, PC-3 and Tsu-Pr1 human prostatic cancer cell lines on the chorio-allantoic membrane. They demonstrated a reliable tumour growth allowing the evaluation of proliferation and apoptosis induction after intravascular or topic application of anticancer drugs[18].

There are very few publications about the use of the CAM assay in human bone or soft tissue sarcoma[19,20]. The literature does not provide any reports that specifically characterise the tumour forming potential of different human osteosarcoma cell lines. Therefore, we established a protocol allowing monitoring of the growth of several osteosarcoma lines on the CAM. The MNNG-HOS, U2OS and SAOS cell lines consistently developed vascularized tumours after 4 days, which then progressively consolidated throughout the growth process. All tumours were derived from established human osteosarcoma cell lines [21-24] and were more or less able to form a solid tumour, but only the MNNG-HOS, SAOS and U2OS reached the consistency as well as average tumour size (Figure 1G) to allow further investigations. The MNNG-HOS cells clearly provided the most reliable and consistent results. The reasons for these differences remain speculative. The difference of up to two million cells between CAM's from different experiments might partly explain the size variances, but it is rather unlikely as this would affect all cell lines. It might be due to the fact that each osteosarcoma cell line produces a differently composed extracellular matrix[21] and thus might have a different potential to degrade the CAM tissue. Possibly only the more aggressive cell lines are able to invade the CAM and induce angiogenesis, which is essential for tumour growth. The MNNG-HOS cell line is derived from HOS cells by treatment with the carcinogenic nitrosamine MNNG[25], and was shown to have a high degree of similarity but also some characteristic differences from the maternal cell line[22] that did not grow well in our model. The fact that a significantly higher mortality rate was seen after grafting of SAOS and U2OS cells (both reliably formed tumours) may be due to tumour cell dissemination in the embryo or secretion of factors which cause blood coagulation. Secretion of such molecules by cancer cells is favoured under metabolic stress such as hypoxia[26], a condition which might well occur during tumour cell implantation on the CAM. Tsuchiya et al.[20] examined 24 human tumour cell lines for their potential to form liver metastases after injecting them into a larger CAM vessel. They have shown that, among others, the MNNG-HOS cells proliferated in the liver. The metastatic potential of osteosarcoma cell lines (143B cells and to a lesser extent MNNG-HOS cells) has also been shown in an orthotopic mouse model[27] as well as in chick embryos after intravascular injection in CAM veins[28]. Thus, this model might also serve to study the development of distant metastases. Pulmonary metastases are very common in osteosarcoma and are the typical cause of death in human[1]. Also speculative at this point, they might also be responsible for the significantly higher death rate in the U2OS and SAOS treated embryos in our study.

The MNNG-HOS cells might be most suitable for further inhibition studies in our model, since this line reliably simulated key features of human osteosarcoma growth such as angiogenesis, necrosis and haemorrhage. Osteoid formation however, did not occur in this model. This might be due to the lack of mature bone in the developing chick embryo or due to the short time span of only 7 days of tumour growth. The experimental osteosarcoma described here are hybrid tumours: they develop out of human cells and secondarily get invaded by newly formed chicken capillaries and stromal cells, therefore allowing further studies aiming at the molecular characterization of osteosarcoma angiogenesis. It might for example be possible to separately study gene expression in the newly formed vessels using Affymetrix chicken GeneChips, in parallel with human GeneChips, which will measure gene expression in the tumour cells. This could lead to the development of new antitumour drugs. The CAM model also is an attractive tool to follow the fate and visualize microscopically the behaviour of the grafted cells[14]. In vivo microscopy is possible at every time point during the growth process, thus allowing direct observations of tumour development and its reactions to intravascular or topical application of anticancer drugs. Additionally, intravital videomicroscopy of the chorioallantoic microcirculation enables studies focussing on metastasis formation. MacDonald et al. used epifluorescence to identify labelled B16F1 melanoma cells, and studied successive stages of metastasis formation in the CAM *in vivo*[29]. This might facilitate the screening of new therapeutic approaches[30] such as molecules targeting the RANK/RANKL/OPG axis[31].

A limit of this model is the short time span of only 7 days for tumour growth which might not have been long enough for the other cell lines to grow to bigger tumours. However, especially this time frame might be of importance for the development of metastases or tumour recurrence. These first steps are heavily dependent on invasion and angiogenesis, which is a promising target for new drugs against solid tumours, including osteosarcoma[32,33].

Our model could serve as a preclinical screening assay for anticancer molecules against osteosarcoma and therefore increase the speed and efficacy of the development of new drugs. More, the model is an excellent alternative to rodent models and fits perfectly into current recommendations of an ethically appreciable use of live animals in cancer research[34].

Last, the experimental tumour model on the CAM might be adapted for other malignant bone and soft-tissue tumours such as chondrosarcoma, Ewing's-sarcoma, malignant fibrous histiocytosis, and benign but locally aggressive lesions such as giant cell tumour of bone.

## Competing interests

The authors declare that they have no competing interests.

## Authors' contributions

MB performed all CAM experiments and drafted the manuscript. AN did all culturing of the cells and helped in drafting of the manuscript. CK and HB did the histology and analysis of the tumours. KA helped with analysis and interpretation of data. MH critically revised the manuscript and together with MB is responsible for the study design. CG and GG participated in the study design and coordination and helped in interpretation of the data. All authors read and approved the final manuscript.
